# Evaluation of Multidrug Efflux Pump Inhibitors by a New Method Using Microfluidic Channels

**DOI:** 10.1371/journal.pone.0018547

**Published:** 2011-04-12

**Authors:** Yoshimi Matsumoto, Kohei Hayama, Shouichi Sakakihara, Kunihiko Nishino, Hiroyuki Noji, Ryota Iino, Akihito Yamaguchi

**Affiliations:** 1 Institute of Scientific and Industrial Research, Osaka University, Ibaraki, Osaka, Japan; 2 Precursory Research for Embryonic Science and Technology, Japan Science and Technology Agency, Chiyoda-ku, Tokyo, Japan; 3 Core Research for Evolutional Science and Technology, Japan Science and Technology Agency, Chiyoda-ku, Tokyo, Japan; 4 Department of Applied Chemistry, University of Tokyo, Bunkyo-ku, Tokyo, Japan; 5 Graduate School of Pharmaceutical Sciences, Osaka University, Suita, Osaka, Japan; University of Birmingham, United Kingdom

## Abstract

Fluorescein-di-β-d-galactopyranoside (FDG), a fluorogenic compound, is hydrolyzed by β-galactosidase in the cytoplasm of *Escherichia coli* to produce a fluorescent dye, fluorescein. We found that both FDG and fluorescein were substrates of efflux pumps, and have developed a new method to evaluate efflux-inhibitory activities in *E. coli* using FDG and a microfluidic channel device. We used *E. coli* MG1655 wild-type, Δ*acrB* (ΔB), Δ*tolC* (ΔC) and Δ*acrB*Δ*tolC* (ΔBC) harboring plasmids carrying the *mexAB-oprM* (pABM) or *mexXY-oprM* (pXYM) genes of *Pseudomonas aeruginosa*. Two inhibitors, MexB-specific pyridopyrimidine (D13-9001) and non-specific Phe-Arg-β-naphthylamide (PAβN) were evaluated. The effects of inhibitors on pumps were observed using the microfluidic channel device under a fluorescence microscope. AcrAB-TolC and analogous pumps effectively prevented FDG influx in wild-type cells, resulting in no fluorescence. In contrast, ΔB or ΔC easily imported and hydrolyzed FDG to fluorescein, which was exported by residual pumps in ΔB. Consequently, fluorescent medium in ΔB and fluorescent cells of ΔC and ΔBC were observed in the microfluidic channels. D13-9001 substantially increased fluorescent cell number in ΔBC/pABM but not in ΔBC/pXYM. PAβN increased medium fluorescence in all strains, especially in the pump deletion mutants, and caused fluorescein accumulation to disappear in ΔC. The checkerboard method revealed that D13-9001 acts synergistically with aztreonam, ciprofloxacin, and erythromycin only against the MexAB-OprM producer (ΔBC/pABM), and PAβN acts synergistically, especially with erythromycin, in all strains including the pump deletion mutants. The results obtained from PAβN were similar to the results from membrane permeabilizer, polymyxin B or polymyxin B nonapeptide by concentration. The new method clarified that D13-9001 specifically inhibited MexAB-OprM in contrast to PAβN, which appeared to be a substrate of the pumps and permeabilized the membranes in *E. coli*.

## Introduction

Increasing multidrug resistance in clinical isolates is currently a major problem in infection control. In particular, the resistance of multidrug resistant *Pseudomonas aeruginosa* (MDRP) to major antipseudomonal agents, such as carbapenems, quinolones, and aminoglycosides [1], [2], has been demonstrated and is known to cause nosocomial outbreaks in Japan [3], [4]. *P. aeruginosa* has natural intrinsic resistance tendencies, and MDRPs have complex resistance mechanisms [5], [6]. In particular, multidrug efflux pumps, especially resistance-nodulation-cell division (RND) family pumps, can decrease the sensitivity of *P. aeruginosa* to various types of compounds [7], [8]. Twelve intrinsic efflux systems belonging to the RND family have been characterized from the genome sequence of *P. aeruginosa* [9], and in particular MexAB-OprM, MexCD-OprJ, MexEF-OprN and MexXY efflux systems are known to have important roles in multidrug resistance [6], [10], [11], [12]. These systems can increase their resistance levels by acquiring additional resistance factors [13], [14]. During this period of new antibacterial agent scarcity, RND pump inhibitors appear useful for treating MDRP infections. The enhancing effects of an experimentally available efflux pump inhibitor, Phe-Arg-β-naphthylamide (PAβN, MC-207,110) [15], on antibacterial activities of compounds in combination with several antibiotics have been published [12], [15], [16], although no clinically useful inhibitor is known. Recently, 3D structures of MexB [17] and co-crystal structures of AcrB with various substrates have been resolved [18], and some information regarding their mechanisms of efflux is available. At present, rational approaches are being employed to develop potent efflux pump inhibitors. However, no satisfactory method to determine the efflux inhibitory activities of candidate compounds directly is available.

**Figure 1:**
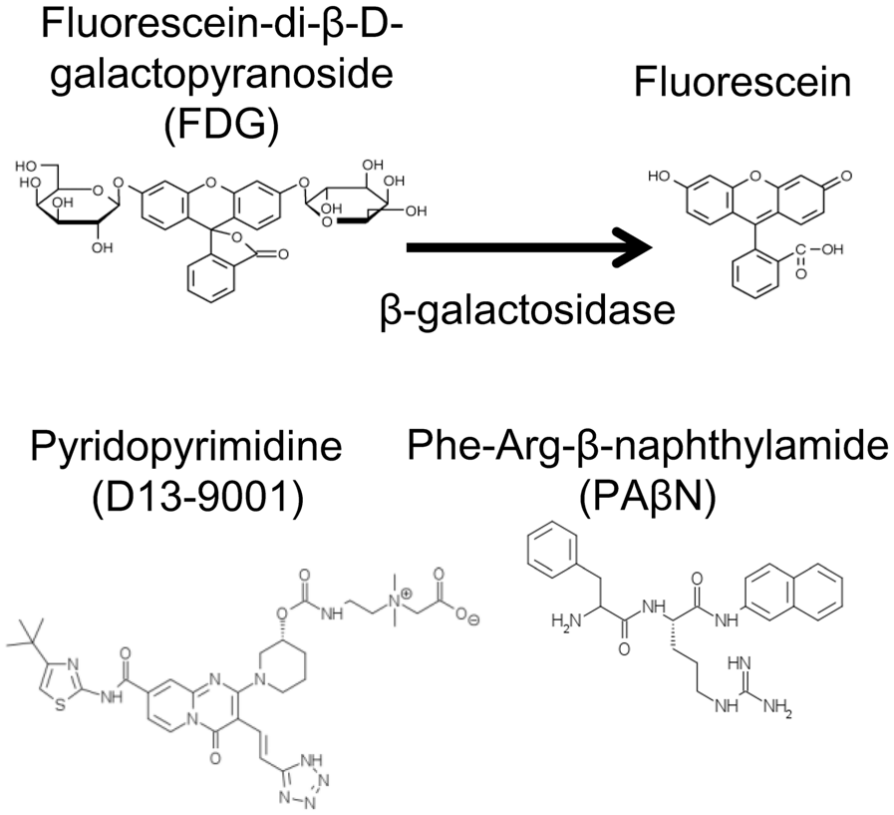
Chemical structures of fluorescein-di-β-d-galactopyranoside (FDG), fluorescein, and inhibitors.

Fluorescein-di-β-d-galactopyranoside (FDG, Figure 1) is a fluorogenic compound that is non-fluorescent until it is hydrolyzed by β-galactosidase in the cytoplasm of *Escherichia coli* to produce a highly fluorescent dye, fluorescein [19], [20], [21]. We first confirmed that both FDG and fluorescein are substrates of RND pumps in *E. coli*. In addition, recent progress in microfabrication technologies including soft lithography [22] has expanded their application in biology [19], [23], [24]. In this study, we constructed a simple microfluidic channel device for bacteria. By combining FDG and the microfluidic device, we developed a novel and highly sensitive method to evaluate the efflux inhibitory activities of compounds against *P. aeruginosa* MexB and MexY in *E. coli*, and clarified difference of action mechanism between two inhibitors, pyridopyrimidine (D13-9001) [25] and PAβN (Figure 1).

## Materials and Methods

### Bacterial strains and plasmids

Table 1 lists all strains and plasmids used in the present study. *E. coli* MG1655 (wild-type), and its deletion mutants—Δ*acrB* (ΔB), Δ*tolC* (ΔC), and Δ*acrB*Δ*tolC* (ΔBC) [26]—were used. The vector plasmid pMMB67HE recombined with efflux pump genes *mexAB-oprM* and *mexXY-oprM* from *P. aeruginosa* [27] were kindly provided by Dr. Taiji Nakae. These plasmids were transformed with ΔBC to construct ΔBC/pMMB67HE (ΔBC/pV), ΔBC/pMMB67HE::*mexAB-oprM* (ΔBC/pABM), and ΔBC/pMMB67HE::*mexXY-oprM* (ΔBC/pXYM). MDRP IMCJ2.S1 (S1) [28] was kindly provided by Dr. Kirikae. *P. aeruginosa* PAO1 was the standard strain.

**Table 1:** Bacterial strains and plasmids.

	Abbreviation	Strain or plasmid	Source or reference
Strain	Wild	*Escherichia coli* MG1655	[26]
	ΔB	MG1655 Δ*acrB*	[26]
	ΔC	MG1655 Δ*tolC*	[26]
	ΔBC	MG1655 Δ*acrB*Δ*tolC*	[26]
	ΔBC/pV	MG1655 Δ*acrB*Δ*tolC*/pMMB67HE	This study
	ΔBC/pABM	MG1655 Δ*acrB*Δ*tolC*/pMMB67HE::*mexAB-oprM*	This study
	ΔBC/pXYM	MG1655 Δ*acrB*Δ*tolC*/pMMB67HE::*mexXY-oprM*	This study
	PAO1	*Pseudomonas aeruginosa* PAO1	
	S1	*Pseudomonas aeruginosa* IMCJ2.S1	[28]
Plasmid	pV	pMMB67HE	[27]
	pABM	pMMB67HE::*mexAB-oprM*	[27]
	pXYM	pMMB67HE::*mexXY-oprM*	[27]

### Antibacterial agents and chemicals

Antibacterials used were aztreonam (ATM; Sigma-Aldrich, Tokyo, Japan), ciprofloxacin (CIP; Tokyo Chemical Industry co., Ltd., Tokyo, Japan), erythromycin (ERY; Nacalai Tesque, Inc., Kyoto, Japan), and polymyxin B (PMB; MERCK KGaA, Darmstadt, Germany). D13-9001 (Daiichi Sankyo Co., Tokyo, Japan; Figure 1) and PAβN (Sigma-Aldrich; Figure 1) were used as efflux pump inhibitors. Polymyxin B nonapeptide (PMBN; Sigma-Aldrich) was used as an outer membrane permeabilizer. Ampicillin (AMP; Sigma-Aldrich) was added to cultures to ensure retention of the plasmid. Lactose (Sigma-Aldrich) or isopropyl-β-d-galactopyranoside (IPTG; Sigma-Aldrich) was added to the medium for β-galactosidase and plasmid-mediated pump induction. FDG (Marker Gene Technologies, Inc., Eugene, USA; Figure 1) and SYTOX Green (Lonza Walkersville, Inc., USA) were used for fluorometry. Nitrocefin (CalBiochem, San Diego, USA) was used as a substrate of β-lactamase in nitrocefin permeabilizing assay.

### Preparation of microfluidic channels

**Figure 2:**
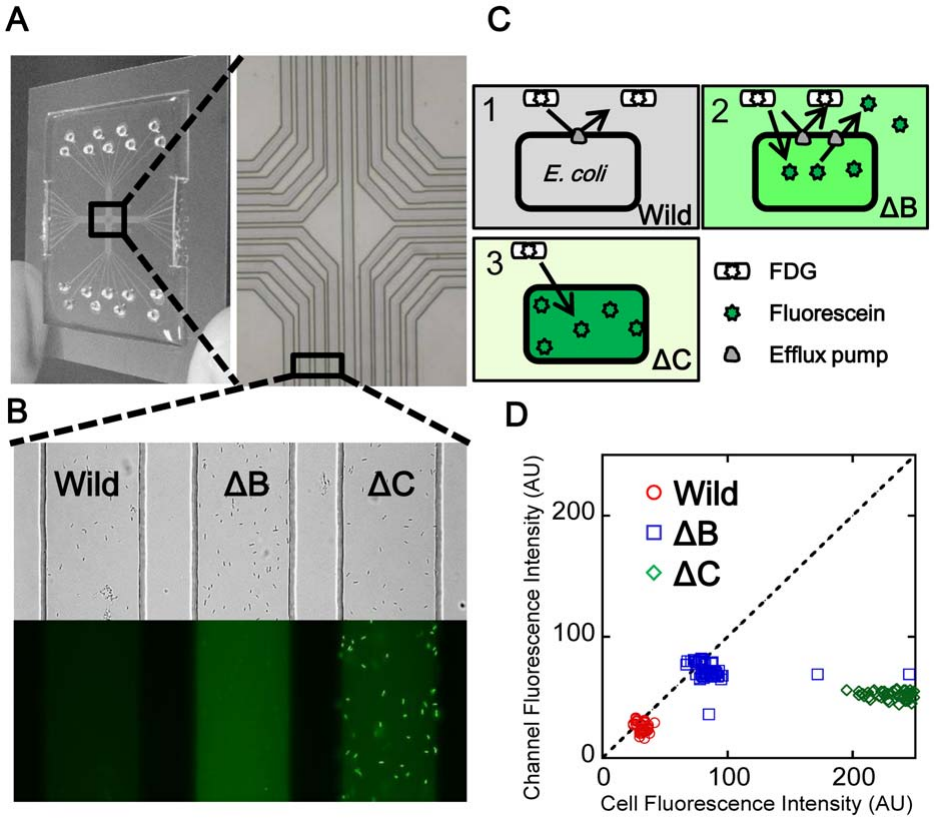
The microfluidic device and principle of the efflux pump inhibition assay used in this study. (A) Images of the microfluidic device used in this study. (B) An example of the assay: Bright-field (top) and fluorescence images (bottom) of the *E. coli* wild-type, ΔB and ΔC cells. The 8-bit gray-scale fluorescence image was displayed using a pseudo-color (black/green). (C) Mechanism of the efflux pump inhibition assay with FDG as a substrate. (D) Distributions of fluorescence intensities of the cells and channels measured in the image shown in (B).

Microfluidic channels (100 µm in width, 17 µm in height, 25–33 mm in length; Figure 2A) fabricated in polydimethylsiloxane (PDMS; Silpot 184, Dow Corning Toray Co., Ltd., Tokyo, Japan) on a cover glass (Matsunami Glass Ind., Ltd., Osaka, Japan) were prepared as follows [22]. The pattern of each channel was designed by a software (Illustrator, Adobe Systems Incorporated, San Jose, USA), and the photomask was prepared by projecting the designed image (demagnified ×10) on a 2.5-inches square glass plate (High precision photo plate PXJP, Konica-Minolta Holdings Inc., Tokyo, Japan). A photoresist (SU8-25, MicroChem, Newton, USA) was spin-coated on a silicon wafer (76 mm in diameter, Ferrotec Co., Tokyo, Japan) at 2000 rpm for 30 s and pre-baked at 65°C for 3 min and 95°C for 3 min on a hot plate (MH-180CS, AS ONE Co., Osaka, Japan). The photoresist was then covered with the photomask and exposed to UV light for 11 s using a mask aligner (ES20, Nanomeric Technology Inc., Tokyo, Japan). The photoresist was baked at 65°C for 1 min and at 95°C for 3 min, after which it was developed for 5 min, and was subsequently hard-baked at 180°C for 1 h. A patterned photoresist was spin-coated with a hydrophobic polymer (0.84% CYTOP, Asahi Glass Co., Ltd., Tokyo, Japan) at 4000 rpm for 30 s, baked at 180°C for 1 h, and used as a mould for the channel. PDMS and a catalyst, mixed (10∶1 by weight) and degassed under vacuum for 30 min, were put in the mould and cured at 100°C for 30 min. Polymerised PDMS peeled off the mould and the cover glass pre-cleaned with ethanol were treated with oxygen plasma using a reactive ion etching instrument (RIE-10NR, Samco, Kyoto, Japan) under appropriate conditions (Oxygen flow: 100 cc/min; Pressure: 50 Pa; RF power: 30 W) for 20 s and then bonded to each other. The inlet and outlet of the channel were then punched (BP-15F, Kai Industries Co., Ltd., Seki, Japan).

### Determination of MICs and checkerboard MICs

MICs of the antibacterial agents and checkerboard MICs of these in combination with the inhibitors or the membrane permeabilizer were determined using the microdilution method defined by the Clinical and Laboratory Standards Institute (CLSI; Wayne, PA, USA). Strains harboring the plasmid were cultured in a medium containing 100 µg/ml AMP and 1 mM IPTG. The best fractional inhibitory concentration (FIC) indices were calculated as [MIC_A in combination_/MIC_A alone_+MIC_B in combination_/MIC_B alon_] Synergy was defined as an FIC index of less than 0.5.

### Efflux pump inhibition assay (FDG assay)

Effects of inhibitors on FDG and fluorescein efflux were observed in the microfluidic channel with a BZ-8000 fluorescence microscope (KEYENCE, Osaka, Japan). Overnight cultures of the strains in Luria–Bertani broth (LB; Becton Dickinson and Company, Sparks, USA) were inoculated in fresh broth and incubated on a shaker until the culture reached an OD_600_ of 0.6–0.8. Lactose (50 mM) was added to induce β-galactosidase in strains harboring no plasmid. LB containing 100 µg/ml AMP and 1 mM IPTG was used to grow strains harboring the plasmid. The cultures were premixed with an inhibitor, injected in the microfluidic channels (Figure 2A) with 100 µg/ml FDG, and observed under a microscope after incubation for 15 min at 37°C. Multiple channels containing different samples were observed simultaneously in a single image field (Figure 2B). The 8-bit gray-scale fluorescence (Figure 2B, bottom) and bright-field (Figure 2B, top) images were sequentially obtained with a cooled CCD camera. Individual cells, most of them are single, adhered to the surface and were immobile in microchannel, were identified by eye and the position of them in fluorescence image was determined manually from the bright-field image. Although it was difficult to discriminate between the intracellular and extracellular fluorescence intensity in the presence of high external fluorescein background due to the limited lateral and longitudinal resolutions of the optical microscope, we were able to correctly determine the position of the cell in the fluorescence image with this procedure. Fluorescence intensity of the individual cells and channels next to the cells were measured using ImageJ software (Figure 2D). The assays were carried out at least two times for individual experimental conditions, and 30–60 cells were analyzed in each experimental condition in a single assay.

### Nitrocefin hydrolysis assay

The wild-type and ΔC strains harbouring plasmid pMMB67HE were constructed and used for the assay. The exponentially growing cultures (OD_600_ 0.6∼0.7) of these strains in LB containing 100 µg/ml of AMP were harvested, and resuspended in AMP-free LB. Nitrocefin was added to the cell suspension to give a final concentration of 50 µg/ml, after 10 min incubation with serial concentrations of PAβN. Hydrolysis of nitrocefin was monitored in a 384-well plate with a SH-8100 microplate reader (Corona Electric Co., Ltd, Hitachinaka, Japan) at 486 nm.

### SYTOX Green uptake assay

Exponentially growing (OD_600_ = 0.7–1.0) wild-type, ΔB, and ΔC cells harvested in PBS containing 0.4% glucose were used. SYTOX Green is a high-affinity nucleic acid stain that easily penetrates cells with compromised membranes, but does not penetrate cells with intact membranes. Membrane integrity was determined after exposure to D13-9001, PAβN, and PMB for 20 min by measuring the uptake of SYTOX Green. Fluorescence was determined (Ex/Em: 504/523 nm) after a 10-min incubation with SYTOX Green in a black 384-well plate with the SH-8100 microplate reader.

## Results

### MICs of antimicrobial agents against tested strains

Table 2 shows MICs of agents against strains of *E. coli* and *P. aeruginosa* used in this study. PAβN had weak antibacterial activity against these *E. coli* and *P. aeruginosa* strains, and its MIC changed by deletion or introduction of the pump genes, which revealed that pump deletion mutants had higher sensitivity to PAβN than the wild-type strain. The results indicate that PAβN is a substrate of RND pumps. D13-9001 had no antibacterial activity against these strains at concentrations less than 64 µg/ml. MIC of ATM against *E. coli* was not influenced by *acrB* and *tolC* deletion or pXYM introduction. However, pABM introduction decreased the sensitivity of ΔBC to ATM by 8-fold. In contrast, MICs of CIP and ERY against *E. coli* were decreased by *acrB* and/or *tolC* deletion and increased by pABM and pXYM introduction. MICs of PMB and PMBN were not influenced by deletion or introduction of these pump genes.

**Table 2:** MICs of antimicrobial agents against tested strains.

Agents	MIC; µg/ml							
	*E. coli*						*P. aeruginosa*	
	Wild	ΔB	ΔC	ΔBC/pV	ΔBC/pABM	ΔBC/pXYM	PAO1	S1
D13-9001	>64	>64	>64	>64	>64	>64	>64	>64
PAβN	256	64	64	32	256	128	512	512
ATM	0.125	0.063	0.125	0.125	1	0.125	2	64
CIP	0.016	0.004	0.002	0.002	0.016	0.031	0.063	32
ERY	32	2	1	1	16	32	256	256
PMB	2	2	2	2	2	2	2	2
PMBN	64	64	64	>8	>8	>8	>8	>8

D13-9001, pyridopyrimidine; PAβN, Phe-Arg-β-naphthylamide; ATM, aztreonam; CIP, ciprofloxacin; ERY, erythromycin; PMB, polymyxin B; PMBN, polymyxin B nonapeptide.

### Combinatorial effects of inhibitors and antibacterial agents in the checkerboard method

**Figure 3:**
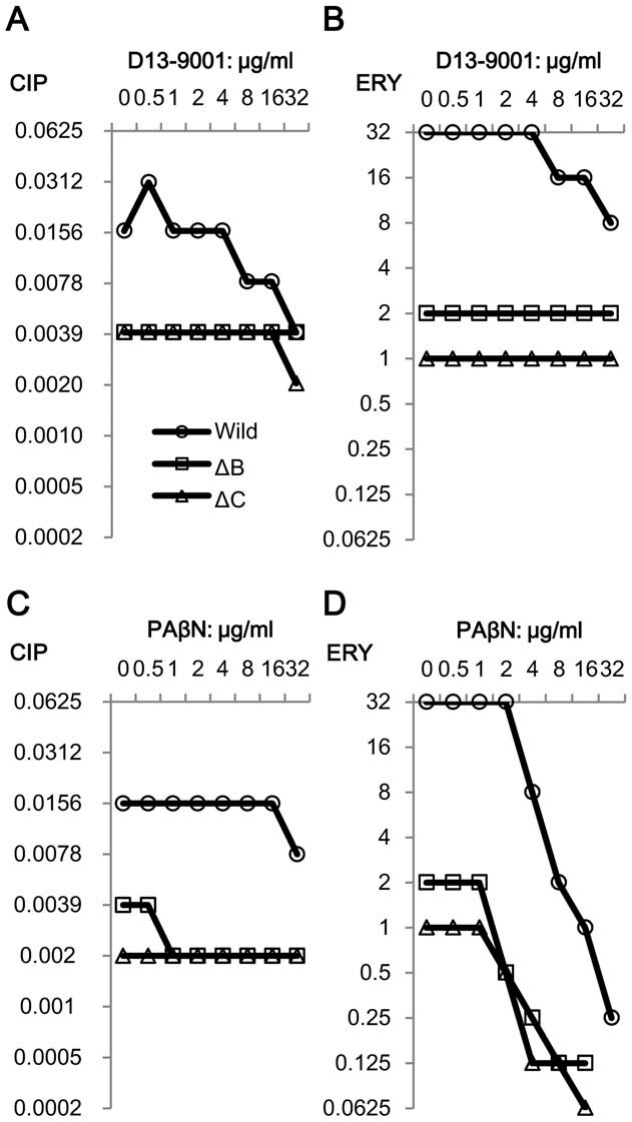
Effects of D13-9001 and PAβN on CIP and ERY activity. *E. coli* MG1655 and its pump deletion mutants were used. Changes in CIP and ERY MICs induced by D13-9001 or PAβN were determined by the checkerboard method.

**Figure 4:**
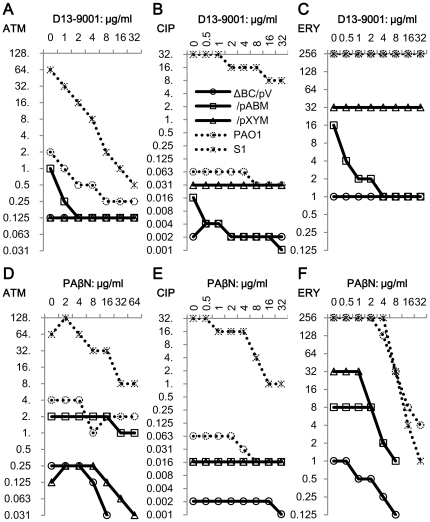
Effect of D13-9001 and PAβN on ATM, CIP, and ERY activity. *E. coli* MG1655 ΔBC expressing MexAB-OprM or MexXY-OprM, and *P. aeruginosa* PAO1 and S1 were used. Changes in ATM, CIP, and ERY MICs induced by D13-9001 or PAβN were determined by the checkerboard method.

We used three different types of antimicrobial agents to evaluate combinatorial effects of inhibitors. ATM is a substrate of MexB, although CIP and ERY are substrates of several pumps. CIP easily penetrates outer membranes of Gram-negative bacteria, but ERY hardly penetrates them [29]. Changes in MIC depending on increased concentrations of inhibitors are shown by each combination of inhibitor and antimicrobial agent. First, the effect of inhibitors on the susceptibilities of *E. coli* MG1655 and its pump deletion mutants, ΔB and ΔC, were compared (Figure 3). D13-9001 acted synergistically with CIP and/or ERY against the wild-type strain and had no effect with either agent against ΔB and ΔC (Figure 3A, B). On the other hand, PAβN increased ERY activity against all strains including ΔB and ΔC (Figure 3D). The lowest FIC index was 0.094 for the wild-type strain and 0.188 for both mutants. In contrast, the effect of PAβN on CIP activity was only additive (FIC index = 0.75–1) against these strains (Figure 3C). Subsequently, the effects of inhibitors on *P. aeruginosa* and *E. coli* expressing pumps from *P. aeruginosa* were examined (Figure 4). D13-9001 acted synergistically with ATM and CIP but not with ERY, which has a higher affinity for MexY than MexB (Table 2), against both sensitive and resistant *P. aeruginosa*, and the synergy between D13-9001 and ATM, which has affinity for MexB but not for MexY, was remarkable in these strains (Figure 4A–C). In *E. coli*, D13-9001 increased the susceptibilities of ΔBC/pABM to all three agents and had no effect on the susceptibilities of ΔBC/pV and ΔBC/pXYM (Figure 4A–C). The lowest FIC indices of PAβN with ATM, CIP, and ERY obtained against MDRP S1 (Figure 4D–F) were 0.188, 0.063, and 0.05, respectively. PAβN again acted synergistically with ERY against all strains including ΔBC/pV. The effect of PAβN was weak on PAO1 with ATM or CIP. Furthermore, the effect of PAβN on CIP activity was not synergistic against all these *E. coli* strains. PAβN acted synergistically with ATM in ΔBC/pXYM and ΔBC/pV, although it was additive in ΔBC/pABM. Synergy between PAβN and these antimicrobial agents was not explainable by efflux pump inhibition by PAβN.

### Inhibitory effect of the efflux pump observed by the FDG assay

At the beginning of development of the FDG assay, we confirmed that induced activities of β-galactosidase in strains used in this study were comparable after acrB and/or tolC deletion and introduction of pumps from *P. aeruginosa* by a plasmid. AcrAB-TolC and the analogous RND pumps effectively prevented FDG influx in *E. coli* wild-type cells, resulting in no fluorescence (Figure 2B, C-1). In contrast, ΔB and ΔC easily infiltrated and hydrolyzed FDG to fluorescein, which accumulated in ΔC cells but not in ΔB cells. Consequently, we observed fluorescent medium in ΔB (Figure 2B, C-2) and fluorescent cells in ΔC (Figure 2B, C-3). Images of these strains in the microfluidic channels are shown in Figure 2B. Fluorescence intensity of each cell identified in a bright-field image was determined and plotted in Figure 2D. Fluorescence distributions of the wild-type, ΔB, and ΔC strains were isolated from each other, and fluorescence was highly accumulated only in ΔC.

**Figure 5:**
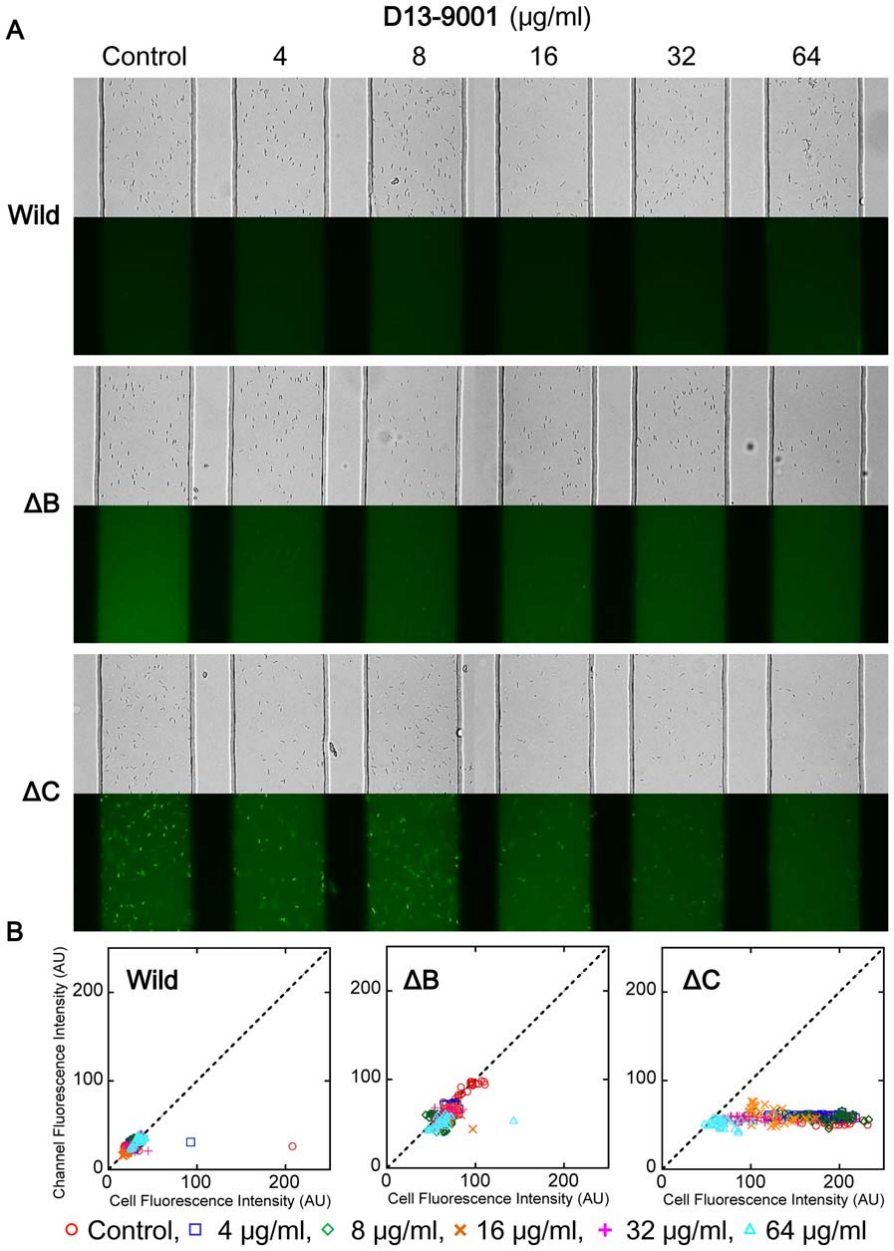
Effects of D13-9001 on FDG degradation determined by the microfluidic channel device. (A) Bright-field (top) and fluorescence images (bottom) of the *E. coli* wild-type, ΔB, and ΔC cells treated with different concentrations of PP. (B) Distributions of the fluorescence intensities of the cells and channels measured in the image shown in (A).

**Figure 6:**
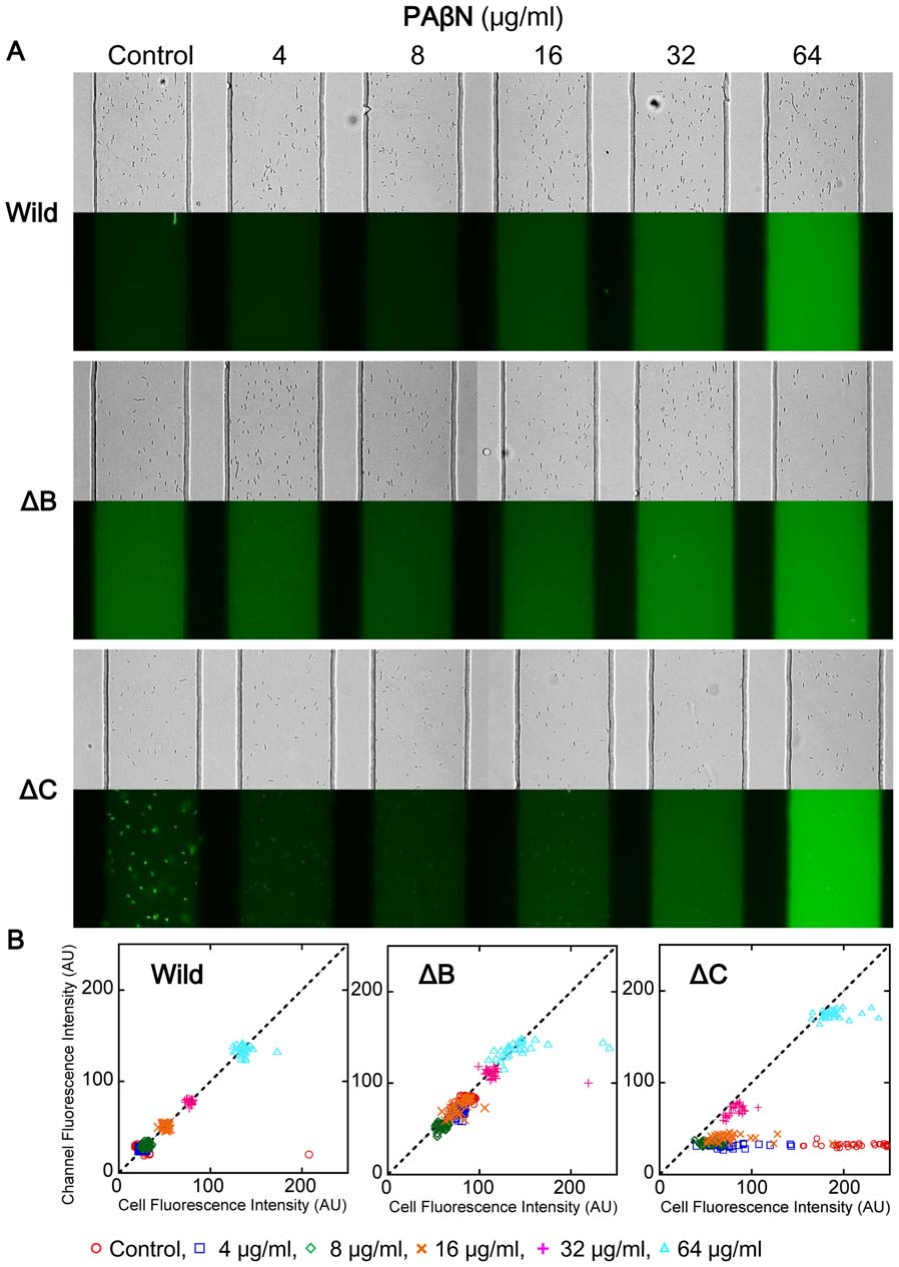
Effects of PAβN on FDG degradation determined by the microfluidic channel device. (A) Bright-field (top) and fluorescence images (bottom) of the *E. coli* wild-type, ΔB, and ΔC cells treated with different concentrations of PAβN. (B) Distributions of the fluorescence intensities of the cells and channels measured in the image shown in (A).

The effect of D13-9001 was not significant in wild-type *E. coli* in the new method (Figure 5). Fluorescence in the device decreased to a minor extent by D13-9001 in a concentration-dependent manner in ΔB and ΔC, and the possibility of FDG blocking activity of D13-9001 was suggested. On the other hand, PAβN increased fluorescence in the medium of all strains, especially in ΔC, and accumulation of fluorescein in the cells of ΔC disappeared (Figure 6).

**Figure 7:**
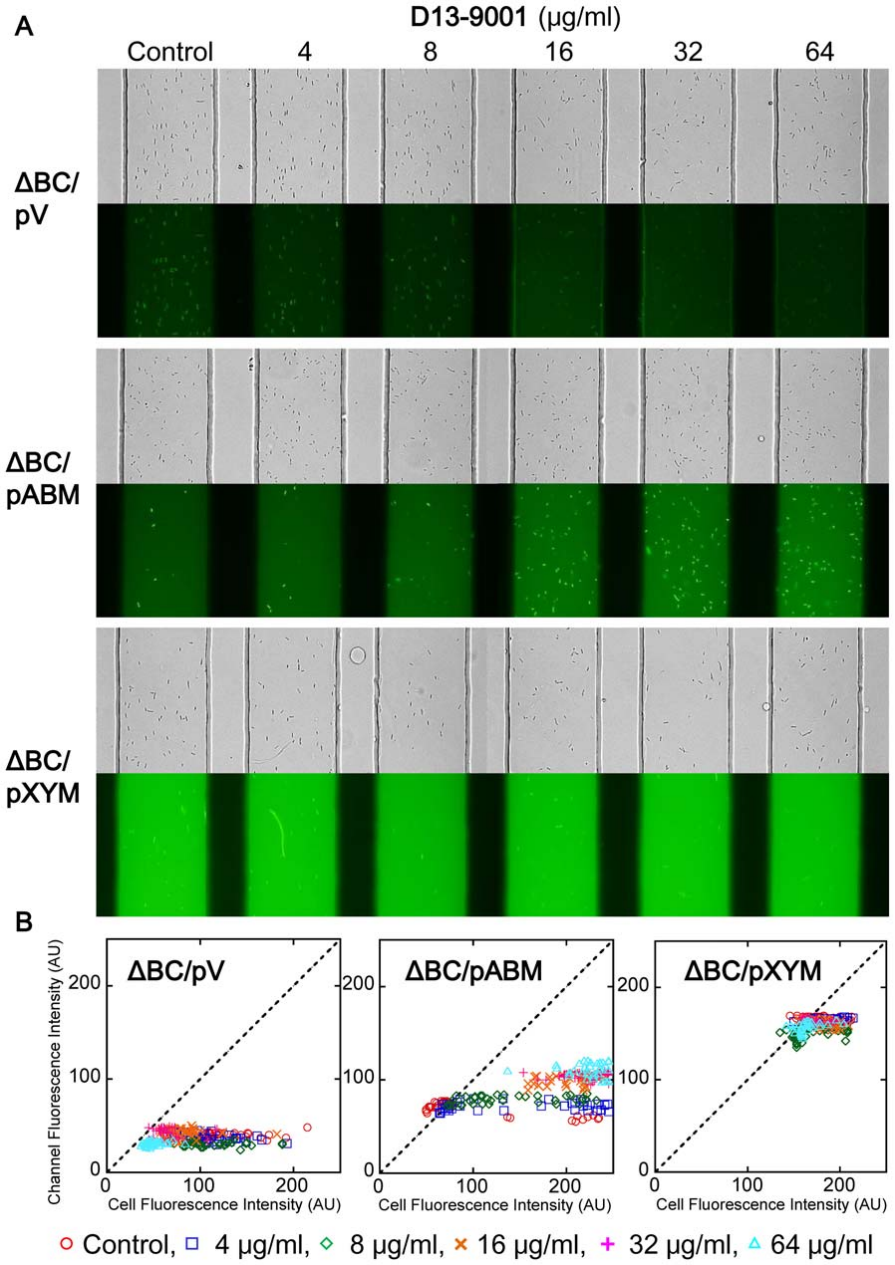
Effect of D13-9001 on MexAB-OprM and MexXY-OprM expressed in *E. coli* MG1655 ΔBC. (A) Bright-field (top) and fluorescence images (bottom) of the ΔBC/pV, ΔBC/pABM, ΔBC/pXYM cells treated with different concentrations of D13-9001. (B) Distributions of the fluorescence intensities of the cells and channels measured in the image shown in (A).

We also evaluated the inhibitors in relation to the MexAB-OprM and MexXY-OprM pumps from *P. aeruginosa* in *E. coli*. The fluorescent medium was observed in ΔBC carrying pABM or pXYM, while fluorescent cells were observed in ΔBC/pV (Figure 7), so these RND pumps from *P. aeruginosa* appeared to be working in *E. coli* ΔBC. D13-9001 clearly increased the accumulation of fluorescein in ΔBC/pABM cells, and fluorescent cells increased by D13-9001 in a concentration-dependent manner in ΔBC/pABM, although it had almost no effect on ΔBC/pXYM. PAβN also increased fluorescence in the medium of all these strains (data not shown).

### Characterization of PAβN activity on membrane in comparison with an outer membrane permeabilizer PMBN

**Figure 8:**
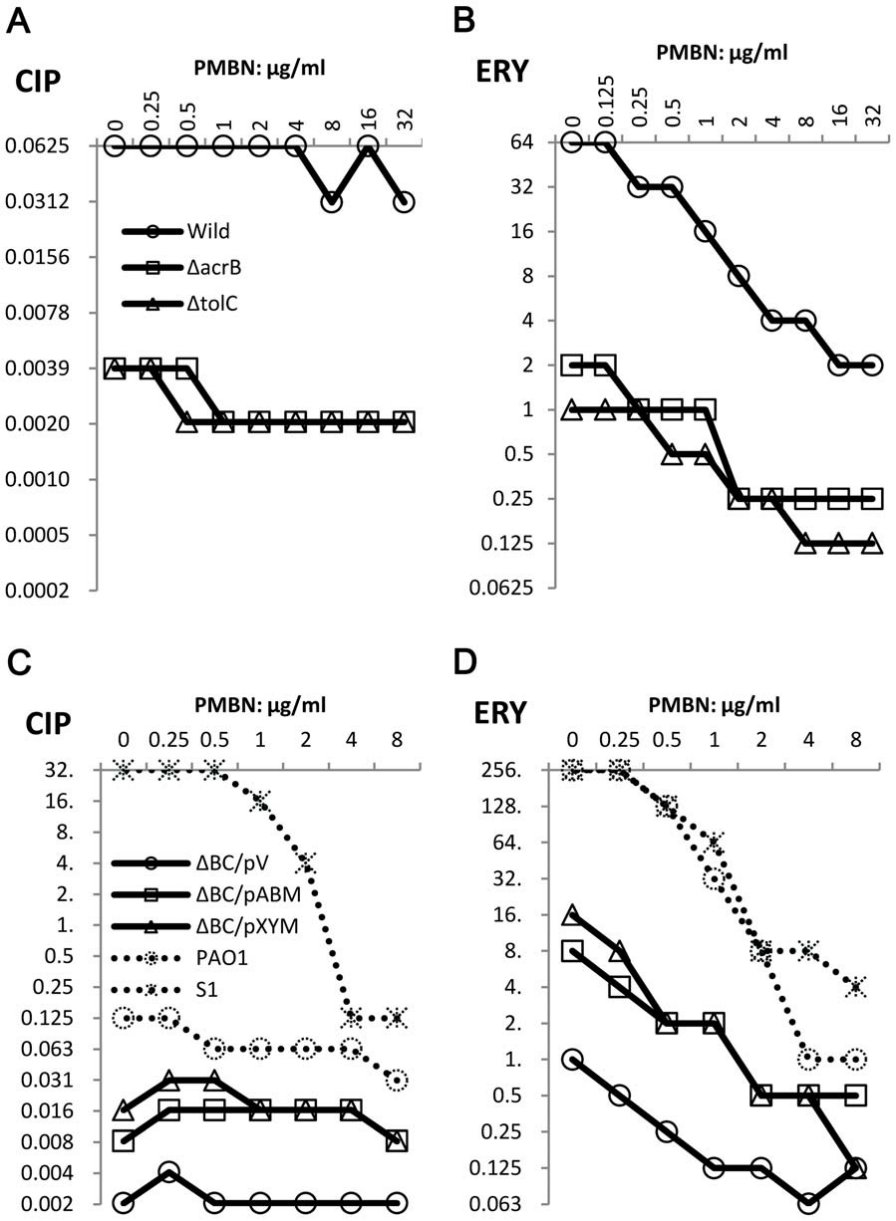
Effect of PMBN on CIP, and ERY activity against *E. coli* and *P. aeruginosa*. Changes in CIP, and ERY MICs induced by PMBN were determined by the checkerboard method.

By the new method, PAβN seemed to have membrane peameabilizing activity. Separately from efflux pump inhibitor, even old, outer membrane permeabilizer has been approached to enhance activity of antimicrobial agents [30], [31], [32], [33]. Among them, PMBN is known to peameabilize outer membrane but inner membrane of Gram-negative bacteria, although PMB peameabilizes both membranes [34], [35], [36]. We determined activity of PMBN in combination with CIP or ERY (Figure 8). Effect of PMBN on CIP activity was additive against the *E. coli* strains and PAO1, and was synergistic against S1. In contrast, PMBN increased ERY activity against all strains including ΔB, ΔC and ΔBC/pV. These results obtained from PMBN were comparable with the results from PAβN. Effect of PMB on ERY was additive against all strains tested (Data not shown).

**Figure 9:**
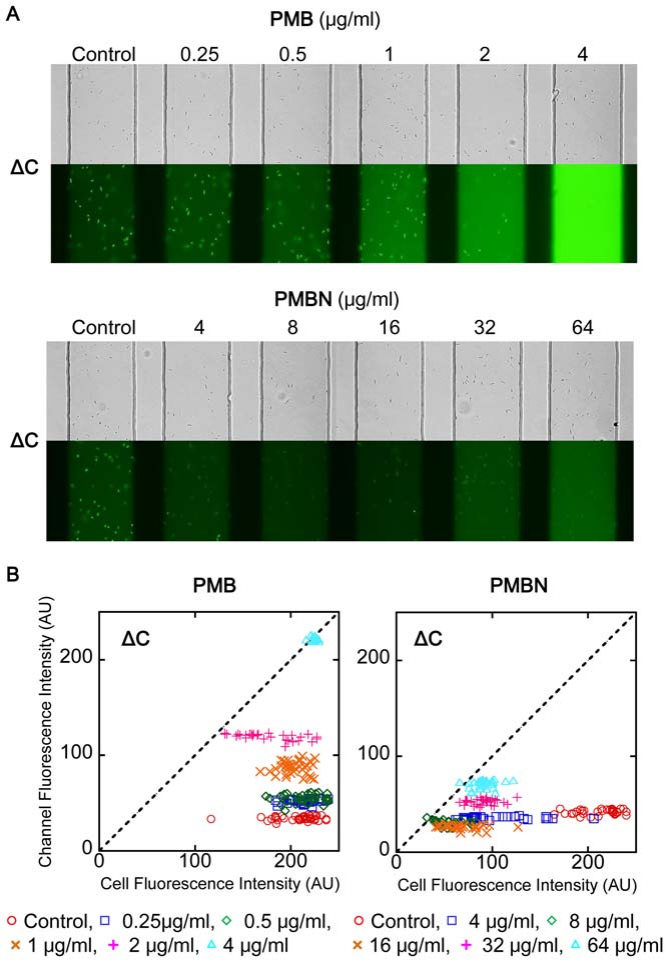
Effects of agents on FDG degradation in Δ*tolC* determined by the microfluidic channel device. (A) Fluorescence images of the *E. coli* ΔC cells treated with different concentrations of PMBN and PMB. (B) Distributions of the fluorescence intensities of the cells and channels measured in the image shown in (A).

We further evaluated membrane peameabilizing activity of PAβN with PMB and PMBN in ΔC by the new device method (Figure 6, Figure 9). Fluorescein accumulation in ΔC cells was disappeared by 4 µg/ml of PAβN as well as by PMBN. PMB increased fluorescence of medium in more than 1 µg/ml, and fluorescein accumulation was still seen in 1 µg/ml of PMB. MexB inhibitor D13-9001 had almost no effect on fluorescein distribution in ΔC (Figure 5). Disappeared accumulation of fluorescein in ΔC cells and increased fluorescence in the medium of ΔC would correspond with peameabilizing of outer membrane and inner membrane of *E. coli*, respectively. The effect of PAβN was similar with PMBN in lower concentrations, and was similar with PMB in higher concentrations (Figure 6). Effect of outer membrane permeabilizer is significant with antibacterial agents that are effectively excluded by the intact outer membrane. Synergistic action of PAβN in combination with ERY seemed come from its outer membrane peameabilizing activity.

### Outer Membrane permeabilization determined by nitrocefin hydrolysis assay

**Figure 10:**
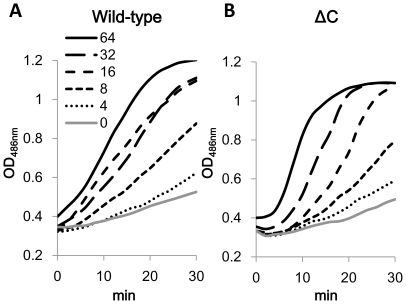
Effect of PAβN on nitrocefin hydrolysis by intact cells of wild-type *E. coli* MG1655 and its *tolC* deletion mutant. Both strains were transformed with pMMB67HE before use. Exponentially growing cells in LB containing 100 µg/ml of AMP were harvested and resuspended in fresh LB for the assay. Nitrocefin hydrolysis was monitored by OD_486 nm_ in a 384-well plate with a plate reader after exposure to PAβN for 10 min. (A) shows results from Wild-type/pV, and (B) shows results from ΔC/pV.

Nitrocefin, a chromogenic β-lactam is easily hydrolyzed by periplasmic β-lactamase of cells having permeabilized outer membrane, so that its increased rate of hydrolysis in intact cells has been used for an indicator of increased outer membrane permeation of nitrocefin [37]. We used both wild-type and ΔC strains harbouring plasmid pMMB67HE for the nitrocefin assay, because nitrocefin is also known as a substrate of AcrB [38] (Figure 10). Ten minutes incubation with PAβN in the concentration higher than 4 µg/ml increased nitrocefin hydrolysis in the both strains. Activity of efflux pumps in the wild-type strain had almost no effect on nitrocefin assay, although MexAB-OprM overproduction in *P. aeruginosa* diminished the effect of PAβN on nitrocefin hydrolysis in the literature of Lomovskaya et al. [15]. The results show stronger activity of PAβN on outer membrane of *E. coli* than on that of *P. aeruginosa* published in the past [15]. Outer membrane permeabilizing activity of PAβN in this nitrocefin hydrolysis assay was harmonized with the activity obtained from the FDG assay.

### Inner Membrane permeabilization determined by SYTOX Green uptake assay

**Figure 11:**
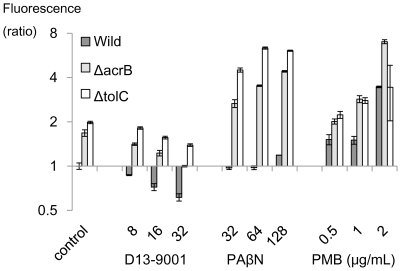
Effect of inhibitors on SYTOX Green uptake in *E. coli* MG1655 and its pump deletion mutants. Exponentially growing cells harvested in PBS containing 0.4% glucose were used. Membrane integrity was determined by measuring SYTOX Green uptake after exposure to D13-9001, PAβN, and PMB for 20 min. Fluorescence was determined (Ex/Em: 504/523 nm) after an incubation of 10 min with SYTOX Green in a black 384-well plate with a plate reader.

SYTOX Green, a DNA intercalater, barely penetrates membranes of intact cells. In the three *E. coli* strains tested, determined fluorescence was slightly higher in pump deletion mutants than in the wild-type strain (Figure 11). The membrane permeabilizer PMB increased SYTOX Green uptake in all strains, regardless of efflux pump activity. PAβN increased SYTOX Green uptake in these strains, although its effect was moderate in the wild-type cells and remarkable in the ΔB and ΔC cells. MICs of PAβN (Table 2) correlated with its inner membrane-damaging activity obtained in Figure 11, which appeared to be the mechanism of its antibacterial action. D13-9001 slightly decreased SYTOX Green uptake in all of the strains as well as the results obtained from FDG uptake in the new method.

## Discussion

**Figure 12:**
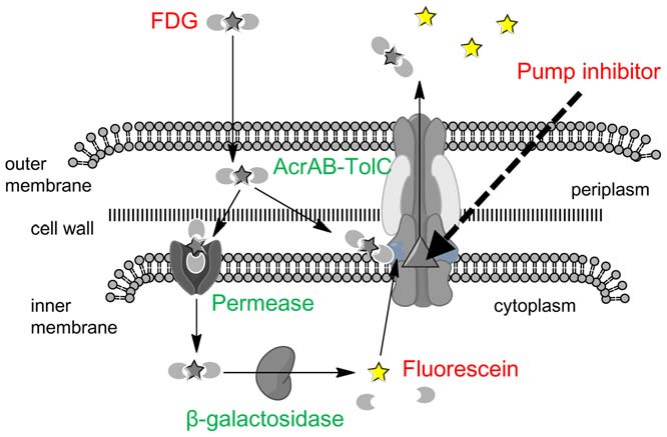
Proposed mechanism of FDG hydrolysis and transport in *E. coli*.

Several fluorometric methods evaluating efflux pump inhibitors have been published using substrates of these pumps such as alanine β-naphthylamide [15], *N*-phenylnaphthylamine [15], ethidium bromide [15], and pyronin Y [39]. Lomovskaya et al. used a related compound MC-002,595 instead of PAβN in the methods using alanine β-naphthylamide or N-phenylnaphthylamine. They could not determine inhibitory activities of PAβN itself by these methods due to the high background fluorescence [15]. Furthermore, PAβN has been reported to have no inhibitory effect on efflux of ethidium bromide [15], [40], [41], which is also known as a substrate of ABC-type transporter [42]. What is more, fluorescence of these compounds was not as strong as that of fluorescein, and thus they are not considered to be suitable for the assay in the microfluidic channel. The role of efflux pumps on FDG hydrolysis in *E. coli* was not fully understood when we chose FDG as a substrate for the assay at the beginning of this study. FDG was defined as a substrate of RND pumps because it was easily hydrolyzed in pump deletion mutants compared with wild-type cells (Figure 2). If FDG was not a substrate of pumps, it will be difficult to discriminate ΔB from wild-type. Fluorescein was also defined as a substrate of pumps from its accumulation in ΔC cells. Furthermore, from the results of complete blockage of FDG hydrolysis by protonophore CCCP addition in all of the strains including ΔC (data not shown), FDG was defined to be actively imported into the cytoplasm. The lactose permease LacY is not a FDG permease because *lacY* deletion from Δ*lacI* mutants (constitutive β-galactosidase producer) had no effect on FDG hydrolysis in the mutants (data not shown), and we have yet to identify a FDG permease. Figure 12 shows the proposed mechanism of FDG hydrolysis in *E. coli*. In wild-type cells, FDG is hardly imported into cytoplasm because FDG is exported by AcrB from periplasm before it is trapped by permease. Rate of FDG influx will increase depending on a concentration of FDG in periplasm until it reach maximum rate. Moderate inhibition of the pumps causes FDG influx and an efflux of fluorescein from the cells by the remaining activity of the pumps, and full inhibition of the pumps results in fluorescein accumulation in cells, similar to what is observed in ΔC cells (Figure 2). A real pump inhibitor without any effect on bacterial membrane will increase fluorescence in wild-type cell by concentration dependent manner and will cause increased accumulation of fluorescence in the cells. In fact, we detected moderately increasing fluorescence and increased accumulation of fluorescence in ΔBC/pABM according to increasing concentration of D13-9001 (Figure 6). In contrast, peameabilization of outer membrane will cause leakage of fluorescein from ΔC cells, and peameabilization of inner membrane will efficiently increase FDG influx and fluorescein production to release it from cells with or without pumps. By the FDG assay, it is easy to detect outer membrane peameabilization by disappearance of fluorescein accumulation in ΔC cells, and inner membrane peameabilization by increased fluorescence especially in the medium of pump deletion mutants. Determination of efflux pump inhibition activity via the typical methods of measuring the influx or efflux of some substrates by their fluorescence with a plate reader makes it difficult to exclude the effect of outer membrane peameabilizing activity. In addition, accurate estimation of FDG hydrolysis through monitoring fluorescence with a plate-reader is impractical because the total fluorescence of fluorescein is higher when diffused in the medium than when accumulated in the cells, and fluorescence determined by a plate reader was usually higher in ΔB than in ΔC. Therefore, fluorescence determined by a plate reader does not accurately correlate with the amount of fluorescein produced, and it is difficult to estimate the inhibitory effect on pumps with FDG by a plate reader. The microfluidic channel method enables discrimination of pure efflux pump inhibition from membrane permeabilization. However, when more than two different pumps are present in a cell, it may be difficult to detect the effect of a specific inhibitor on either of them. To overcome this problem, we need a mutant deleting all RND pumps and sustaining TolC in which fluorescein is accumulated. Deletions of *acrB*, *acrD*, *acrEF*, *mdtABC*, and *mdtEF* were not sufficient to ensure accumulation of fluorescein in a manner similar to that in ΔC (data not shown).

D13-9001 is known to specifically inhibit the MexAB-OprM pump [25]. In the microfluidic channel method, D13-9001 clearly inhibited the MexAB-OprM pump but did not inhibit the MexXY-OprM pump (Figure 7). The effect of D13-9001 on AcrAB-TolC of *E. coli* would be detected by this method when a strain producing only AcrAB-TolC is available. Combinatorial effects of D13-9001 with the antimicrobials assessed by the checkerboard method are easily understandable by the inhibition of the AcrB or MexB pump in all the tested strains of *E. coli* and *P. aeruginosa*. The synergy of D13-9001 was determined to be the strongest with ATM, which is a substrate of MexB and not of other pumps.

Throughout this study, we could not definitively answer the question as to whether PAβN really acts on efflux pumps. We observed that PAβN increased FDG hydrolysis and leakage of fluorescein in all strains, especially in pump deletion mutants, by this new method (Figure 6). Since PAβN was first reported, it has been universally recognized as an efflux pump inhibitor [12], [15], and the effect of PAβN on MDRP S1 was remarkably synergistic with all the agents examined in this study (Figure 4). However, PAβN increased the susceptibilities of pump deletion mutants of *E. coli* especially to ERY, although it had almost no effect on CIP against *E. coli* expressing MexAB-OprM or MexXY-OprM (Figure 4E), which means PAβN could not inhibit efflux of CIP by MexB or MexY. PAβN also increased susceptibility of *E. coli* expressing MexXY-OprM to ATM, which is not a substrate of MexXY-OprM (Figure 4D). These actions of PAβN do not correlate with efflux pumps, and are similar to those of outer membrane permeabilizer PMBN. The effect of PAβN on the outer membrane was already known when it was discovered [15]. They used nitrocefin hydrolysis assay by intact cells to evaluate outer membrane permeabilizing activity of PAβN in *P. aeruginosa*, and permeabilizing activity was visible in lower than 16 µg/ml on outer membrane of a pump deficient strain or a CCCP applied strain. Nitrocefin is a substrate of efflux pumps [38], so that permeabilizing activity should be evaluated in pump deficient condition. Permeabilizing activity of PAβN on outer membrane of *E. coli* by the assay using nitrocefin (Figure 10) was comparable to the activity obtained from the FDG assay and was higher than that on outer membrane of *P. aeruginosa* [15]. Outer membrane permeabilizing activity of PAβN have also been published by other researchers using resazurin as a substrate [43]. In our FDG assay, the effect of PAβN on the outer membrane of *E. coli* was visible in 4 µg/ml (Figure 6). LB was used for the FDG assay and the results would be easy to compare with the results of synergy in MICs with antimicrobial agents. LB used in our study contains 0.24 mM Mg^2+^ (information from the manufacturer). When additional 1 mM Mg^2+^ was added to LB, effect of PAβN on outer membrane decreased to some extent although it was not disappeared in the FDG assay (data not shown), and accordingly, addition of Mg^2+^ to the medium also decreased synergistic activity of PAβN with ERY to *P. aeruginosa* PAO1 (data not shown). Outer membrane permeabilizing activity is known to increase the sensitivity of bacteria to antimicrobial agents like ERY which excluded by the intact outer membrane [32], [44] as well as efflux pump inhibition, and we could get similar results by PMBN. The effect of PAβN on antimicrobial agents (Figure 3–4) seems to be explainable with its activity on membranes of *E. coli*. We could not obtain any clear evidence of the inhibitory activity of PAβN on the efflux pump itself. Although competitive inhibition of efflux pumps by another substrate was not proved in *E. coli* by Elkins, et al. [45], the possibility that PAβN competes with a substrate for binding to the efflux pump cannot be excluded completely. Precise mechanism of synergy between PAβN and antimicrobial agents should be elucidated elsewhere.

Nevertheless, susceptibility-augmenting agents such as PAβN may be useful in combination with a substrate of multi efflux pumps such as ERY and CIP because it will be difficult to develop a super inhibitor that can inhibit all pumps including those with different structures. The effect of D13-9001 is simple and specific to strains expressing AcrB or MexB pumps, and D13-9001 shows the most synergistic effect with ATM, which is the substrate preferred by MexB and no other pumps. From this new method, it is concluded that D13-9001 specifically inhibits MexB, in contrast to PAβN, which appeared to be a substrate of the pumps and permeabilized the membranes of *E. coli*.

## References

[1] Kirikae T, Mizuguchi Y, Arakawa Y (2008). Investigation of isolation rates of Pseudomonas aeruginosa with and without multidrug resistance in medical facilities and clinical laboratories in Japan.. J Antimicrob Chemother.

[2] Sekiguchi J, Teruya K, Horii K, Kuroda E, Konosaki H (2007). Molecular epidemiology of outbreaks and containment of drug-resistant Pseudomonas aeruginosa in a Tokyo hospital.. J Infect Chemother.

[3] Sekiguchi J, Asagi T, Miyoshi-Akiyama T, Kasai A, Mizuguchi Y (2007). Outbreaks of multidrug-resistant Pseudomonas aeruginosa in community hospitals in Japan.. J Clin Microbiol.

[4] Satoh R, Tsukada H, Tanabe Y, Tamura Y, Yamamoto T (2008). An outbreak and isolation of drug-resistant Pseudomonas aeruginosa at Niigata University Hospital, Japan.. J Infect Chemother.

[5] Livermore DM (2002). Multiple mechanisms of antimicrobial resistance in Pseudomonas aeruginosa: Our worst nightmare?. Clinical Infectious Diseases.

[6] Lister PD, Wolter DJ, Hanson ND (2009). Antibacterial-resistant Pseudomonas aeruginosa: clinical impact and complex regulation of chromosomally encoded resistance mechanisms.. Clin Microbiol Rev.

[7] Ryan BM, Dougherty TJ, Beaulieu D, Chuang J, Dougherty BA (2001). Efflux in bacteria: what do we really know about it?. Expert Opin Investig Drugs.

[8] Masuda N, Sakagawa E, Ohya S, Gotoh N, Tsujimoto H (2000). Substrate specificities of MexAB-OprM, MexCD-OprJ, and MexXY-oprM efflux pumps in Pseudomonas aeruginosa.. Antimicrob Agents Chemother.

[9] Schweizer HP (2003). Efflux as a mechanism of resistance to antimicrobials in Pseudomonas aeruginosa and related bacteria: unanswered questions.. Genet Mol Res.

[10] Llanes C, Hocquet D, Vogne C, Benali-Baitich D, Neuwirth C (2004). Clinical strains of Pseudomonas aeruginosa overproducing MexAB-OprM and MexXY efflux pumps simultaneously.. Antimicrob Agents Chemother.

[11] Morita Y, Kimura N, Mima T, Mizushima T, Tsuchiya T (2001). Roles of MexXY- and MexAB-multidrug efflux pumps in intrinsic multidrug resistance of Pseudomonas aeruginosa PAO1.. J Gen Appl Microbiol.

[12] Mesaros N, Glupczynski Y, Avrain L, Caceres NE, Tulkens PM (2007). A combined phenotypic and genotypic method for the detection of Mex efflux pumps in Pseudomonas aeruginosa.. J Antimicrob Chemother.

[13] Henrichfreise B, Wiegand I, Pfister W, Wiedemann B (2007). Resistance mechanisms of multiresistant Pseudomonas aeruginosa strains from Germany and correlation with hypermutation.. Antimicrob Agents Chemother.

[14] Giske CG, Buaro L, Sundsfjord A, Wretlind B (2008). Alterations of porin, pumps, and penicillin-binding proteins in carbapenem resistant clinical isolates of Pseudomonas aeruginosa.. Microb Drug Resist.

[15] Lomovskaya O, Warren MS, Lee A, Galazzo J, Fronko R (2001). Identification and characterization of inhibitors of multidrug resistance efflux pumps in Pseudomonas aeruginosa: novel agents for combination therapy.. Antimicrob Agents Chemother.

[16] Tohidpour A, Peerayeh SN, Mehrabadi JF, Yazdi HR (2009). Determination of the Efflux Pump-Mediated Resistance Prevalence in Pseudomonas aeruginosa, Using an Efflux Pump Inhibitor.. Current Microbiology.

[17] Sennhauser G, Bukowska MA, Briand C, Grutter MG (2009). Crystal Structure of the Multidrug Exporter MexB from Pseudomonas aeruginosa.. Journal of Molecular Biology.

[18] Murakami S, Nakashima R, Yamashita E, Matsumoto T, Yamaguchi A (2006). Crystal structures of a multidrug transporter reveal a functionally rotating mechanism.. Nature.

[19] Russo-Marie F, Roederer M, Sager B, Herzenberg LA, Kaiser D (1993). Beta-galactosidase activity in single differentiating bacterial cells.. Proc Natl Acad Sci U S A.

[20] Yang NC, Hu ML (2004). A fluorimetric method using fluorescein di-beta-D-galactopyranoside for quantifying the senescence-associated beta-galactosidase activity in human foreskin fibroblast Hs68 cells.. Anal Biochem.

[21] Fieldler F, Hinz H (1994). No intermediate channelling in stepwise hydrolysis of fluorescein di-beta-D-galactoside by beta-galactosidase.. Eur J Biochem.

[22] Whitesides GM, Ostuni E, Takayama S, Jiang XY, Ingber DE (2001). Soft lithography in biology and biochemistry.. Annual Review of Biomedical Engineering.

[23] Weibel DB, Diluzio WR, Whitesides GM (2007). Microfabrication meets microbiology.. Nat Rev Microbiol.

[24] Lenshof A, Laurell T (2010). Continuous separation of cells and particles in microfluidic systems.. Chem Soc Rev.

[25] Yoshida K, Nakayama K, Ohtsuka M, Kuru N, Yokomizo Y (2007). MexAB-OprM specific efflux pump inhibitors in Pseudomonas aeruginosa. Part 7: highly soluble and in vivo active quaternary ammonium analogue D13-9001, a potential preclinical candidate.. Bioorg Med Chem.

[26] Nishino K, Senda Y, Yamaguchi A (2008). The AraC-family regulator GadX enhances multidrug resistance in Escherichia coli by activating expression of mdtEF multidrug efflux genes.. J Infect Chemother.

[27] Mokhonov VV, Mokhonova EI, Akama H, Nakae T (2004). Role of the membrane fusion protein in the assembly of resistance-nodulation-cell division multidrug efflux pump in Pseudomonas aeruginosa.. Biochem Biophys Res Commun.

[28] Sekiguchi J, Asagi T, Miyoshi-Akiyama T, Fujino T, Kobayashi I (2005). Multidrug-resistant Pseudomonas aeruginosa strain that caused an outbreak in a neurosurgery ward and its aac(6′)-Iae gene cassette encoding a novel aminoglycoside acetyltransferase.. Antimicrob Agents Chemother.

[29] Vaara M (1993). Outer membrane permeability barrier to azithromycin, clarithromycin, and roxithromycin in gram-negative enteric bacteria.. Antimicrob Agents Chemother.

[30] Vaara M (1992). Agents that increase the permeability of the outer membrane.. Microbiol Rev.

[31] Vaara M, Porro M (1996). Group of peptides that act synergistically with hydrophobic antibiotics against gram-negative enteric bacteria.. Antimicrob Agents Chemother.

[32] Vaara M, Siikanen O, Apajalahti J, Fox J, Frimodt-Moller N (2010). A novel polymyxin derivative that lacks the fatty acid tail and carries only three positive charges has strong synergism with agents excluded by the intact outer membrane.. Antimicrob Agents Chemother.

[33] Vingsbo Lundberg C, Vaara T, Frimodt-Moller N, Vaara M (2010). Novel polymyxin derivatives are effective in treating experimental Escherichia coli peritoneal infection in mice.. J Antimicrob Chemother.

[34] Vaara M, Vaara T (1983). Polycations as outer membrane-disorganizing agents.. Antimicrob Agents Chemother.

[35] Vaara M, Vaara T (1983). Polycations sensitize enteric bacteria to antibiotics.. Antimicrob Agents Chemother.

[36] Viljanen P, Vaara M (1984). Susceptibility of gram-negative bacteria to polymyxin B nonapeptide.. Antimicrob Agents Chemother.

[37] Hancock RE, Wong PG (1984). Compounds which increase the permeability of the Pseudomonas aeruginosa outer membrane.. Antimicrob Agents Chemother.

[38] Nagano K, Nikaido H (2009). Kinetic behavior of the major multidrug efflux pump AcrB of Escherichia coli.. Proc Natl Acad Sci U S A.

[39] Kaatz GW, Moudgal VV, Seo SM, Kristiansen JE (2003). Phenothiazines and thioxanthenes inhibit multidrug efflux pump activity in Staphylococcus aureus.. Antimicrob Agents Chemother.

[40] Viveiros M, Martins A, Paixao L, Rodrigues L, Martins M (2008). Demonstration of intrinsic efflux activity of Escherichia coli K-12 AG100 by an automated ethidium bromide method.. International Journal of Antimicrobial Agents.

[41] Schumacher A, Steinke P, Bohnert JA, Akova M, Jonas D (2006). Effect of 1-(1-naphthylmethyl)-piperazine, a novel putative efflux pump inhibitor, on antimicrobial drug susceptibility in clinical isolates of Enterobacteriaceae other than Escherichia coli.. Journal of Antimicrobial Chemotherapy.

[42] Martins A, Spengler G, Rodrigues L, Viveiros M, Ramos J (2009). pH Modulation of efflux pump activity of multi-drug resistant Escherichia coli: protection during its passage and eventual colonization of the colon.. PLoS One.

[43] Vidal-Aroca F, Meng A, Minz T, Page MGP, Dreier J (2009). Use of resazurin to detect mefloquine as an efflux-pump inhibitor in Pseudomonas aeruginosa and Escherichia coli.. Journal of Microbiological Methods.

[44] Vaara M (2009). New approaches in peptide antibiotics.. Curr Opin Pharmacol.

[45] Elkins CA, Mullis LB (2007). Substrate competition studies using whole-cell accumulation assays with the major tripartite multidrug efflux pumps of Escherichia coli.. Antimicrobial Agents and Chemotherapy.

